# EM-AUC: A Novel Algorithm for Evaluating Anomaly Based Network Intrusion Detection Systems

**DOI:** 10.3390/s25010078

**Published:** 2024-12-26

**Authors:** Kevin Z. Bai, John M. Fossaceca

**Affiliations:** 1 Independent Researcher, Westwood, MA 02090, USA; 2 Department of Engineering Management and Systems Engineering, George Washington University, Washington, DC 20052, USA; jfossaceca@gwu.edu

**Keywords:** network intrusion detection, unsupervised machine learning models, EM-AUC algorithm, missing data inference, Area Under the Roc Curve, Area Under the Precision-Recall Curve

## Abstract

Effective network intrusion detection using anomaly scores from unsupervised machine learning models depends on the performance of the models. Although unsupervised models do not require labels during the training and testing phases, the assessment of their performance metrics during the evaluation phase still requires comparing anomaly scores against labels. In real-world scenarios, the absence of labels in massive network datasets makes it infeasible to calculate performance metrics. Therefore, it is valuable to develop an algorithm that calculates robust performance metrics without using labels. In this paper, we propose a novel algorithm, Expectation Maximization-Area Under the Curve (EM-AUC), to derive the Area Under the ROC Curve (AUC-ROC) and the Area Under the Precision-Recall Curve (AUC-PR) by treating the unavailable labels as missing data and replacing them through their posterior probabilities. This algorithm was applied to two network intrusion datasets, yielding robust results. To the best of our knowledge, this is the first time AUC-ROC and AUC-PR, derived without labels, have been used to evaluate network intrusion detection systems. The EM-AUC algorithm enables model training, testing, and performance evaluation to proceed without comprehensive labels, offering a cost-effective and scalable solution for selecting the most effective models for network intrusion detection.

## 1. Introduction

Unsupervised machine learning models have been widely studied for network intrusion detection. Unlike supervised learning, unsupervised learning does not require explicit labels; instead, it focuses on identifying patterns within normal data. Anomalous data points that significantly deviate from these learned patterns can then be detected.

Although unsupervised models do not need labels during the training and testing phases, their accuracy and performance assessment in the evaluation phase still require comparing predictions against labels. For network intrusion detection, acquiring labeled network traffic data through manual labeling by network experts can be time-consuming and costly. Alternatively, automatic labeling in simulated environments can generate large volumes of network traffic data. However, both manual and simulated labels are often not considered the gold standard due to human error or system limitations. Additionally, the rapid evolution of malicious behavior poses challenges in maintaining and updating these labeled datasets, resulting in their quick obsolescence. Given these challenges, it is worthwhile developing an algorithm to assess the performance of unsupervised network intrusion detection models when labels are unavailable or imperfect during the evaluation phase.

In this paper, we propose an Expectation Maximization-Area Under the Curve (EM-AUC) algorithm to treat the unavailable labels or imperfect labels as missing data and replace them through their posterior probabilities. Zhou et al. [1] used an Expectation Maximization (EM) based technique for estimating model performance in the absence of labels in medical diagnosis. We further developed the EM technique to estimate additional performance metrics, such as the Area Under the Precision-Recall Curve (AUC-PR), alongside the Area Under the Receiver Operating Characteristic Curve (AUC-ROC). This allows us to assess the performance of a set of unsupervised network intrusion detection models without the need for labels during the evaluation phase. Our proposed system is a cost-effective and scalable solution for network intrusion detection, as it does not require labels for training, testing, and evaluation, thereby completely eliminating the need to label and update large volumes of network traffic data.

In this paper, Section 2 provides a review of related research. Section 3 outlines our proposed methodology in detail. Section 4 discusses our experimental setup and presents the results of model performance, while Section 5 concludes the paper with a discussion.

## 2. Related Studies

Obtaining labeled data for network intrusion detection presents several challenges [2]. Notably, accessing real network traffic data is often impractical, especially in systems with critical functions, or when the data contains sensitive information [3]. Many publicly available datasets for network intrusion detection are simulated to replicate real-world network traffic. Unfortunately, this simulated approach can obscure key details for differentiating normal from malicious behavior. Automated labeling in simulations, designed to reduce the cost and time of manual labeling, often produces inaccurate labels without adequate human oversight [4]. Another challenge with labeled data is the difficulty of keeping it up to date. Specifically, these datasets are typically static and quickly become outdated, failing to reflect emerging threats or zero-day attacks that have not occurred previously [5].

To address these challenges, unsupervised machine learning models can be utilized when labels are unavailable or inaccurate. Typical unsupervised learning models can be categorized into clustering, neighbor-based, density-based, statistical, angle-based, and classification-based methods [6]. The most representative unsupervised learning models include K-means [7], K-Nearest Neighbor [8], Local Outlier Factor [9], Histogram-Based Outlier Score [10], Angle-Based Outlier Detection [11], One-Class Support Vector Machine [12,13], Isolation Forest [14], and Autoencoders [15,16,17]. Multiple studies have tested specific unsupervised models for network intrusion detection. Portnoy et al. [18] introduced a clustering-based unsupervised network intrusion detection model that effectively detects a wide range of intrusion types while maintaining low false positive rates. Aminanto and Kim [19] introduced a method that employs an unsupervised stacked autoencoder for feature extraction and a K-means clustering algorithm for classification. Truong-Huu et al. [20] conducted an empirical study to explore the effectiveness of Generative Adversarial Networks (GANs) in detecting network anomalies. Binbusayyis and Vaiyapuri [21] presented a novel unsupervised intrusion detection method that combines a one-dimensional convolutional autoencoder with a one-class support vector machine. Dutta et al. [22] have also proposed an intrusion detection mechanism that utilizes Deep AutoEncoder and multiple Deep Decoders for unsupervised classification.

Furthermore, comparative studies of multiple unsupervised anomaly detection methods across different network datasets have been conducted to evaluate the effectiveness of these algorithms. Falcão et al. [6] performed a comprehensive evaluation of twelve unsupervised learning models for anomaly-based intrusion detection using five datasets. Meira et al. [23] conducted an empirical study comparing six unsupervised models for detecting unknown attacks in two datasets. Ding et al. [24] compared four widely used novelty detection methods across ten benchmark datasets that vary in complexity. Goldstein and Uchida [25] compared nineteen unsupervised anomaly detection models on ten datasets for various use cases. Zoppi et al. [26] addressed this by evaluating seventeen unsupervised models with eleven different datasets.

In all the previous research mentioned above and most recent studies we surveyed [27,28,29,30,31,32,33,34,35,36], the performance of these techniques was evaluated based on labeled data. Obtaining accurately labeled data is challenging in practice, which creates a need for methods to evaluate unsupervised learning techniques without relying on labels. Umemneku Chikere et al. [37] systematically reviewed the approaches developed to evaluate the performance of diagnostic methods in the absence of accurate labels or ground truth. These approaches can be classified into ad-hoc methods and model-based methods, which assume the true status is either unobserved or treated as a latent variable. Specific model-based approaches include the Multivariate Normal Mixture Latent Model, Random Effects Model, and Bayesian approaches. Zhou et al. [1] proposed an EM based technique for estimating ROC curves in the absence of labels in medical diagnosis.

The EM-based method [1] can be extended to assess the performance of anomaly-based network intrusion detection models without requiring labels, which is the focus of this paper. The EM approach proposed by Zhou et al. [1] provides only the AUC-ROC metric. In our study, we expand its capabilities to estimate additional performance metrics, such as AUC-PR, alongside AUC-ROC. To the best of our knowledge, this is the first study in which both AUC-ROC and AUC-PR are algorithmically derived through missing data inference without using labels. Moreover, the derived AUC-PR can be adopted as an effective performance metric for anomaly detection with class imbalance, as it considers the tradeoff between precision and recall. Compared to the alternative methods reviewed in [37], our EM-AUC approach is a latent class model that does not rely on specific modeling assumptions and provides explicit closed-form solutions. It is easier to implement because of the clear closed-form solution and is more robust, as it does not depend on specific assumptions. In our study, the performance metrics derived by algorithms without using labels provide a new perspective for evaluating classifiers, which is crucial in fields where labels are challenging to define or obtain.

## 3. Methodology

**Figure 1:**
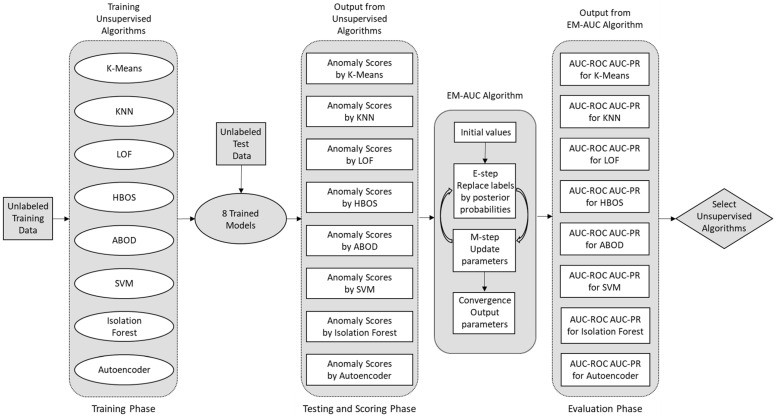
EM-AUC based system for evaluating unsupervised models.

In this paper, we experimentally evaluate a pool of eight widely used one-class unsupervised anomaly detection models on the NSL-KDD [38,39] and UNSW-NB15 [40,41] datasets by EM-AUC algorithm. For one-class anomaly detection, only normal traffic records are used for training. In real-world scenarios, labeled attack records are scarce, while normal traffic data is abundant and readily available. Once the model is trained using normal data, it can be used to detect anomalies and emerging zero-day attacks in the test data. Each unsupervised machine learning model employed in this paper produces an anomaly score for each traffic record in the test data. Our proposed EM-AUC algorithm is then used to take anomaly scores as the sole input, treating the unavailable labels as missing data and replacing them through their posterior probabilities. This enables us to derive AUC-ROC and AUC-PR to evaluate the performance of the eight one-class models without requiring the labels. The diagram in Figure 1 illustrates the workflow of the proposed system during the training, testing, and performance evaluation phases.

### 3.1. One-Class Network Intrusion Detection Models

This section describes the mechanism for generating anomaly scores from each of the one-class network intrusion detection models. The anomaly scores, as the sole input, are then fed into the EM-AUC algorithm for model evaluation and selection, without using labels, in the next phase.

#### 3.1.1. K-Means Clustering (K-Means)

The K-Means algorithm groups data points into *k* clusters [42]. In the one-class k-means approach, the algorithm is trained exclusively on normal data. Initially, *k* centroids are randomly initialized. Each normal data point is then assigned to the nearest centroid using a distance metric. After all normal data points have been assigned, the centroids are updated to be the mean of the data points in each cluster. This process iterates until the centroids converge or a predetermined number of iterations is reached.

During testing, each point in the test set is assigned to the nearest cluster centroid obtained from the training phase, and the anomaly score for each test point is derived based on its distance to the nearest centroid [43]. Points that are significantly far from any centroid are considered anomalous, as abnormal data points typically do not fit well into the clusters formed by the normal data.

#### 3.1.2. K-Nearest Neighbor (KNN)

K-Nearest Neighbor (KNN) assumes that normal data points cluster together in feature space, while anomalies are more distant. The algorithm first memorizes all normal traffic in the training set. During testing, the distance metric from each test data point to its nearest neighbors in the training set is calculated. In this research, *k* equals 1, so the anomaly score is simply the distance from a data point to its nearest neighbor [44].

#### 3.1.3. Local Outlier Factor (LOF)

In the One-Class Local Outlier Factor (LOF) algorithm, the anomaly score measures how a test point’s local density compares to its nearest neighbors, highlighting deviations from normal density patterns. The algorithm identifies each test point’s k-nearest neighbors from the training set. To understand how far each point has to reach to connect with its neighbors, a sequence of distance-based measurements is used. To assess the reachability distance between two points s and t, the direct distance between s and t denoted as $dist\left(s,t\right)$ is compared with the distance from s to its *k*th nearest neighbor denoted as ${dist}_{k}\left(s\right)$. The reachability distance ${reach-dist}_{k}\left(s,t\right)$ is defined as the greater of these two values by Equation (1).

(1) $${reach-dist}_{k}\left(s,t\right)=\max\left(dist\left(s,t\right),{dist}_{k}\left(s\right)\right)$$

Local Reachability Density (LRD) is defined as the reciprocal of this average reachability distance for point s by Equation (2).

(2) $${LRD}_{k}\left(s\right)=\frac{1}{{average-reach-dist}_{k}\left(s\right)}$$

A low average reachability distance indicates that s is in a dense region, resulting in a high LRD. The anomaly score is then computed as a ratio using the test point’s LRD and the average LRD of its k-nearest neighbors from the training set [10]. This anomaly score assesses how the local density of the test point deviates from the density patterns observed in the normal data.

#### 3.1.4. Histogram-Based Outlier Score (HBOS)

The Histogram-based Outlier Score (HBOS) is a statistical approach that uses histograms to detect anomalies in a dataset. During training, a histogram is created for each feature based on its values from all normal instances. Essentially, a histogram splits the range of values into intervals and counts the number of values within each interval [45].

During testing, for each feature j of a data point x, the probability density $p_{j}(x_{j}$) is estimated based on the histogram created from normal training data. To calculate $p_{j}(x_{j}$), the number of data points that fall into the bin containing $x_{j}$ is divided by the total number of data points in the dataset. The anomaly score for data point x is computed by Equation (3).

(3) $$Score(x)=\frac{1}{f}\sum_{j=1}^{f}{-log(p}_{j}\left(x_{j}\right))$$

Here, f represents the number of features. The negative log likelihood ${-log(p}_{j}(x_{j}))$ can be interpreted as the anomaly score for each feature, where lower density values indicate a higher likelihood of being an anomaly.

#### 3.1.5. Angle-Based Outlier Detection (ABOD)

Angle-Based Outlier Detection (ABOD) is a technique used to identify anomalies by analyzing the angles between data points. During training, ABOD is configured using data consisting of normal samples. It calculates the angles between each data point and its neighbors to establish the typical angle distribution for normal traffic. During testing, angles are calculated for each point in the test dataset, which includes both normal and abnormal instances. To determine if a data point is an anomaly, its angles with its neighbors are compared to the normal patterns learned during training. Data points that exhibit significant deviations from typical angles and have low variance are flagged as anomalies [11]. In particular, normal traffic typically receives a lower anomaly score because it shows high angle variance due to complex geometric relationships within a dense cluster. In contrast, anomalous traffic is assigned a higher anomaly score since it is far from the normal cluster and exhibits low variance due to its more uniform angular relationships.

#### 3.1.6. One-Class Support Vector Machine (One-Class SVM)

A One-Class Support Vector Machine (SVM) is an unsupervised learning technique for anomaly detection, trained on data from only the normal class. It learns a decision boundary that encloses most normal data points in the feature space. The training data is often transformed into a higher-dimensional space using a kernel function for more effective boundary learning. A slack parameter can be defined to balance between including more points within the boundary and allowing some to be outside of it. If a kernel function is used, test data is also transformed into this high-dimensional space during testing, ensuring consistent application of the boundary. The model scores each test data point based on its distance from the boundary to determine how well it fits within the learned region [12]. Data points with a high anomaly score are far away and outside the boundary, indicating a higher likelihood of being anomalies.

#### 3.1.7. Isolation Forest (IF)

The One-Class Isolation Forest (IF) algorithm trains multiple Isolation Trees using normal traffic. To build each tree, a feature and a split value are randomly chosen to divide the data into two subsets. This process is repeated recursively for each subset until a data point is isolated in a leaf node or the tree reaches a maximum depth to prevent overfitting. When evaluating test data points, the algorithm calculates the path length needed to isolate each point using the trained Isolation Trees. The average path length across all trees for each data point is then determined and used to derive the anomaly score [14]. Since anomalies are typically distinct or less common compared to normal data, they are more easily isolated. Therefore, data points with shorter average path lengths will have higher anomaly scores, indicating a greater likelihood of being anomalies.

#### 3.1.8. Autoencoder (AE)

The One-Class Autoencoder (AE) is an artificial neural network specifically designed for unsupervised learning, comprising both an encoder and a decoder [46]. The encoder maps the input data to a lower-dimensional space, while the decoder attempts to reconstruct the original input from this compact representation. In this research, the autoencoder is trained exclusively on normal traffic to learn how to compress and reconstruct these normal instances with minimal error. During the testing phase, the trained autoencoder processes the test data points through both the encoder and decoder to assess how well it can reconstruct them. The anomaly score is calculated as the error between the original test data point and its reconstructed version. Since the autoencoder was trained solely on normal traffic, it is expected to have difficulty reconstructing anomalous data points, leading to higher reconstruction errors for those points.

### 3.2. EM-AUC Algorithm

#### 3.2.1. AUC Based on Gold Standard

When gold standard or perfect labels are available, they can be used to calculate AUC-ROC and AUC-PR to assess the effectiveness of the anomaly scores produced by the unsupervised models. For a given threshold of the anomaly scores, a confusion matrix can be derived as in Table 1.

Subsequently, the True Positive Rate (TPR), False Positive Rate (FPR), Precision and Recall can be calculated for a specific threshold by Equations (4)–(6).

(4) $$TPR\;\left(Recall\right)=\frac{TP}{TP+FN}$$

(5) $$FPR=\frac{FP}{FP+TN}$$

(6) $$Precision=\frac{TP}{TP+FP}$$

The ROC curve is generated by plotting the true positive rate against the false positive rate for each threshold [47]. AUC-ROC can be interpreted as the model’s ability to correctly identify traffic records on a scale from 0.5 to 1, where 1 is best and 0.5 is as good as a random guess.

Another important metric is AUC-PR, which is calculated as the area under the precision-recall curve. AUC-PR indicates whether the model can correctly identify all the intrusion examples without accidentally marking too many normal examples as intrusions. Thus, AUC-PR is high when the model can effectively detect intrusions. A random classifier has an AUC-PR equal to the percentage of intrusions [48]; for example, 0.15 if there are 15% intrusion examples in the data. A perfect classifier has an AUC-PR of 1.0.

Single TPR and FPR values are limited as they reflect performance at a specific threshold and do not reflect the trade-off between TPR and FPR. AUC-ROC is more informative, as it evaluates model performance across all thresholds, providing an aggregated measure of the model’s ability to distinguish between positive and negative classes while balancing true positives and false positives. Similarly, AUC-PR summarizes model performance across all thresholds as well. When class imbalance is present, AUC-PR is preferred over AUC-ROC.

Since the labels are available in both datasets used in this study, the AUC-ROC and AUC-PR can be calculated based on these labels, as described in this section. To assess the effectiveness of our proposed EM-AUC, we compared the AUC-ROC and AUC-PR based on the labels with those derived from the proposed EM-AUC. The details of this comparison are summarized in the results section.

#### 3.2.2. EM-AUC Algorithm

In real-world scenarios, the absence of labels in massive network data makes it impossible to calculate performance metrics such as AUC-ROC and AUC-PR. Even when labels are obtained, inaccurate labels due to potential human error or system limitations can lead to an overestimation or underestimation of the performance metrics. Hence, in this study, we use the EM-AUC algorithm to compute model performance metrics such as AUC-ROC and AUC-PR. In the EM-AUC algorithm, the complete data includes both the observed anomaly scores and the unobserved true intrusion status. The true intrusion status $M_{i}$ of each traffic record is assumed to be unknown and treated as missing data. The complete data log-likelihood can be written by Equation (7).

(7) $$l_{c}=\sum_{i=1}^{N}\left[M_{i}\log p_{1}g_{1}\left(y_{i}\right)+\left(1-M_{i}\right)\log p_{0}g_{0}\left(y_{i}\right)\right]$$

where $p_{1}$ is the intrusion prevalence, and $p_{0}=1-p_{1}$; $y_{i}=\left(y_{i11,}\ldots ,y_{i1J},\ldots ,y_{iK1},\ldots ,y_{iKJ}\right)$, a K×J vector consists of binary variables $y_{ikj}$, defined as $y_{ikj}=1$ if the *i*th traffic record’s *k*th anomaly score is *j*, and is zero otherwise; $g_{m}\left(y_{i}\right)$ is the conditional probability that the *i*th traffic record’s score vector is $y_{i}$ given the true intrusion status $M_{i}$, i.e., $g_{m}\left(y_{i}\right)=P\left(y_{i}|M_{i}=m\right)$. In other words, the above complete data log-likelihood function includes both the unobserved true intrusion status $M_{i}$ and the observed anomaly scoring data $y_{i}$.

The EM-AUC algorithm consists of two steps: the E-step and the M-step. In the E-step, the true intrusion status (i.e., perfect labels) $M_{i}$ are treated as missing data and replaced with their probabilities when calculating the conditional expectation of the complete data log-likelihood. The probabilities are typically initialized using a random variable generator and updated during the M-step. As a result, the labels are no longer needed. In the M-step, the key parameter estimates required for computing AUC-ROC and AUC-PR are updated by maximizing the conditional expectation of the complete log-likelihood. The key parameters include the intrusion prevalence $p_{1}\;and$ $\psi_{mkj}$, which represents the probability of the *k*th algorithm scoring *j* given the record’s true status. This iterative process continues until convergence, optimizing the parameter estimates to fit the observed data.

More specifically, after the key parameters $p_{1}$ and $\psi_{mkj}$ are obtained, the false positive rate and true positive rate for the *k*th machine learning model and cut-off *j* can be defined by Equations (8) and (9). The false positive rate is the summation of $\psi_{0kl}$, interpreted as the probability of *k*th algorithm scoring greater than *j* given the record’s true status is normal. Similarly, the true positive rate is the summation of $\psi_{1kl},$ interpreted as the probability of the *k*th algorithm scoring greater than *j* given the record’s true status is intrusion.

(8) $$FPR_{k}\left(j\right)=P\left(S_{ik}\geq j|M_{i}=0\right)=\sum_{l=j}^{J}\psi_{0kl}$$

(9) $$TPR_{k}\left(j\right)=P\left(S_{ik}\geq j|M_{i}=1\right)=\sum_{l=j}^{J}\psi_{1kl}$$

The ROC curve for the *k*th unsupervised model is obtained by connecting the coordinate pairs of (FPR, TPR). Similarly, the precision-recall curve is constructed by plotting the coordinate pairs (recall, precision), as shown in Equations (10) and (11). The area under the curves can be calculated using the trapezoidal rule.

(10) $${Recall}_{k}\left(j\right)=TPR_{k}\left(j\right)$$

(11) $${Precision}_{k}\left(j\right)=\frac{\sum_{l=j}^{J}\psi_{1kl}p_{1}}{\sum_{l=j}^{J}\psi_{1kl}p_{1}+\sum_{l=j}^{J}\psi_{0kl}{(1-p}_{1})}$$

**Figure 2:**
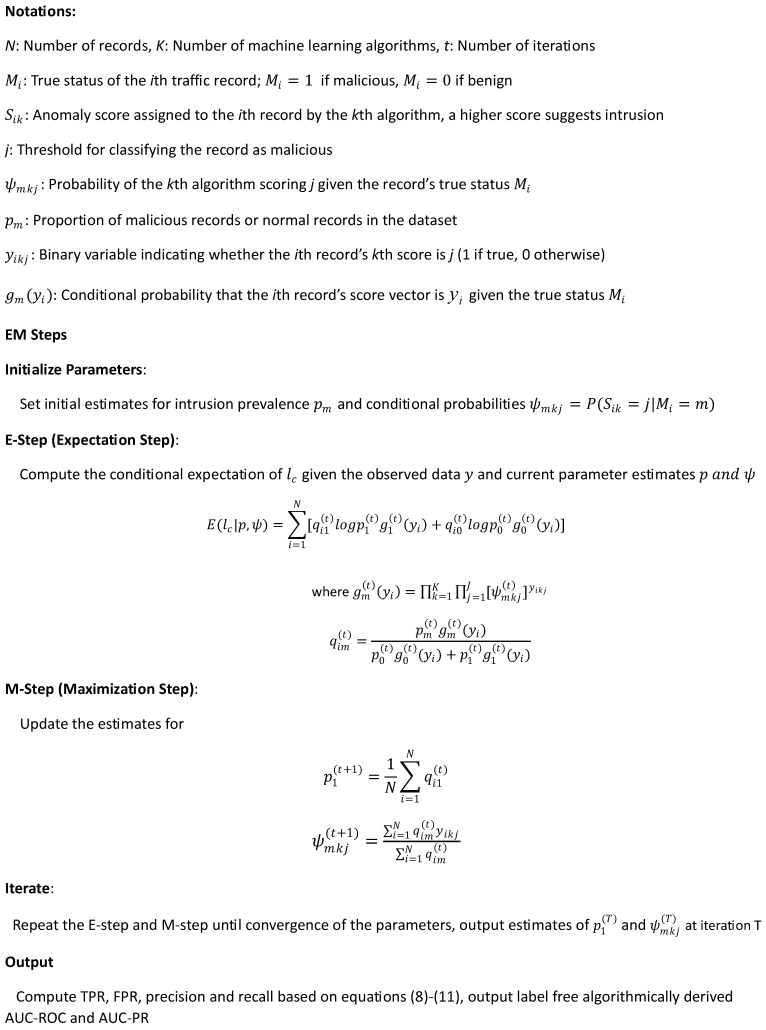
EM-AUC algorithm.

The details of the EM-AUC algorithm, along with notations, are described in Figure 2.

## 4. Experiments and Results

### 4.1. Datasets

In this study, the NSL-KDD and UNSW-NB15 datasets were chosen for the experiments. The KDDTrain+ dataset contains 125,973 samples, 41 features, and five classes, including a normal class and various attack classes (i.e., DoS, probe, R2L, and U2R). The scope of this research is to evaluate unsupervised learning techniques for binary outcomes, and the multi-class classification is not the focus of this study. The data distribution by intrusion status for the KDDTrain+ dataset is summarized in Table 2.

Similarly, the data distribution by intrusion status for the UNSW_NB15_training-set is summarized in Table 3. The UNSW_NB15_training-set contains 175,341 samples, 42 features, and 10 classes, including a normal class and various attack classes (i.e., Backdoor, Analysis, Fuzzers, Shellcode, Reconnaissance, Exploits, DoS, Worms, and Generic). Both the UNSW_NB15_training-set and the KDDTrain+ dataset were created from simulated environments to facilitate the evaluation of intrusion detection systems. The simulated labels in both datasets are generally not considered perfect labels or gold standard labels due to limitations in the simulation and labeling processes.

The two datasets used in this research do not have the class imbalance issue between the normal and intrusion classes, as the intrusion records have already been oversampled in both datasets. In the NSL-KDD dataset, the ratio of normal to intrusion records is 1.1:1, while in the UNSW-NB15 dataset, it is 0.47:1, where the number of intrusion records exceeds the number of normal records.

### 4.2. Experiments

Before the unsupervised models were trained, data pre-processing steps were performed for the KDDTrain+ dataset and the UNSW_NB15_training-set separately. Certain features were eliminated because either they have no variation among the classes or their information is irrelevant for anomaly detection. We retained the majority of the features for training to minimize the loss of relevant information. Continuous variables were standardized using the min-max technique to transform each continuous variable’s range to be between 0 and 1. Both normalization and min-max standardization were considered. Since the min-max transformation does not assume any specific distribution, we chose it for data preprocessing. Categorical variables were transformed using one-hot encoding to create dummy indicators, with the first level serving as the reference level.

To conduct the experiments, each dataset was randomly split into training and test sets, each consisting of 50% of the original data. This random data split was performed five times on the original dataset, resulting in five sets of training and test data for both the KDD study and the UNSW study. For each training set, only normal traffic records were retained. Specifically, eight one-class models, including K-means, KNN, LOF, HBOS, ABOD, IF, SVM, and Autoencoder, were trained using the training set containing only normal traffic records. Due to the absence of perfect data labels or a definitive ground truth in the real world, determining the optimal hyperparameter values is not feasible. As a result, hyperparameters are either set to their default values or adjusted based on domain knowledge. In this research, hyperparameters were set to their default values provided by Python packages with version of 3.8.5.

During the test stage, the models were tested on the test set, which includes both normal and attack records. Each testing record was assigned an anomaly score by each of the eight unsupervised models. The performance of the eight sets of anomaly scores was measured using AUC-ROC and AUC-PR, which are derived from the EM-AUC algorithm without using labels. The experiments were repeated five times on the five sets of training and test data for both the KDD study and the UNSW study to avoid bias. For easier implementation, each set of anomaly scores produced by an unsupervised model was first converted to a 10-point scale based on quantiles and then fed into the EM-AUC algorithm as input. During the implementation of the EM-AUC algorithm in R, multiple sets of initial values were explored to ensure a broad coverage of possible starting points. For each set of initial values, the parameter estimates over iterations were plotted to assess convergence. It was observed that each parameter estimate converged quickly.

### 4.3. Results

#### 4.3.1. NSL-KDD Results

**Figure 3:**
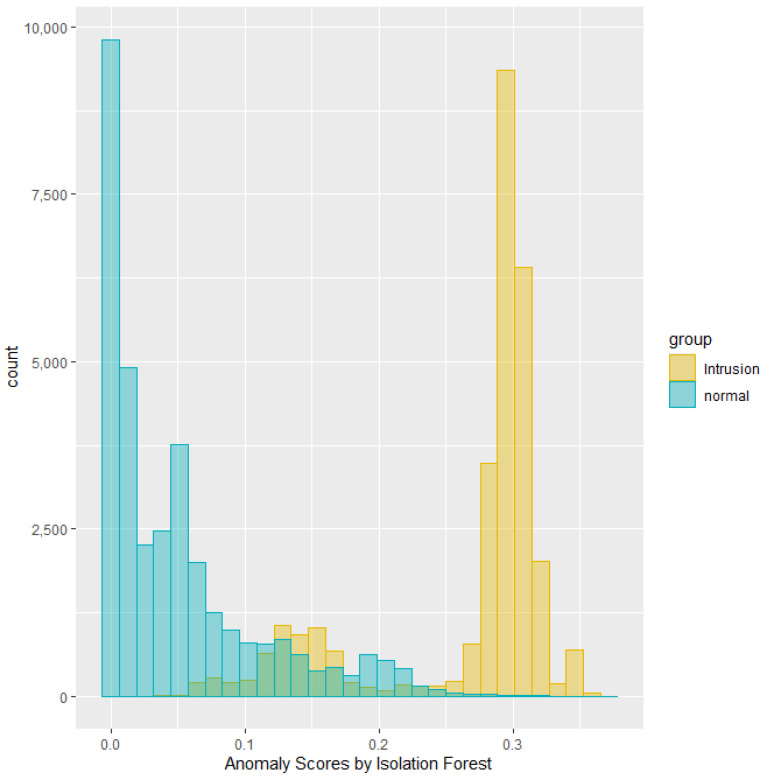
Histogram of anomaly scores produced by Isolation Forest.

This section summarizes the results from running experiments on the NSL-KDD dataset. Anomaly scores can demonstrate overlap between two classes in their distribution. For instance, the histogram in Figure 3 depicts the distribution of anomaly scores generated by the Isolation Forest, grouped by the intrusion labels for a specific test dataset. The green area represents the distribution of anomaly scores for the labeled normal traffic, while the yellow area represents the distribution for the labeled intrusion traffic. Notably, the scores indicate a separation between normal and intrusion traffic with some overlap.

**Figure 4:**
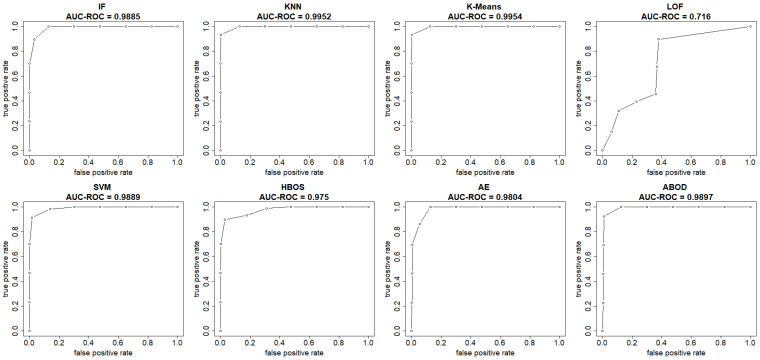
ROC curves (FPR vs. TPR) estimated by EM-AUC for NSL-KDD dataset from one experiment.

**Figure 5:**
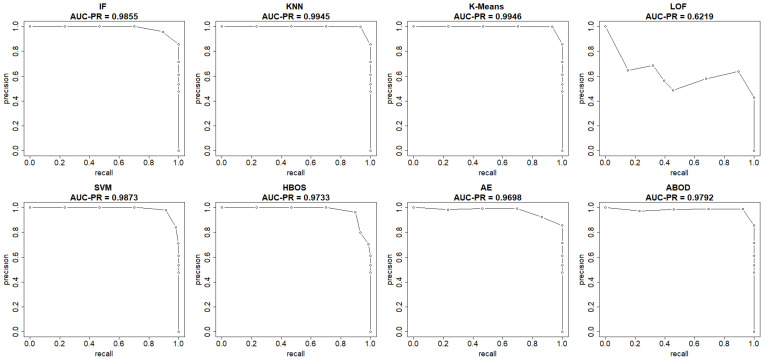
Precision-Recall curves (recall vs. precision) estimated by EM-AUC for NSL-KDD dataset from one experiment.

The plots in Figure 4 and Figure 5 show both the ROC curve and the precision-recall curve, estimated using the EM-AUC algorithm, for each unsupervised learning model from an experiment of a single random data split. In Figure 4, the *x*-axis represents the false positive rates, and the *y*-axis represents the true positive rates at different thresholds of the anomaly scores. Each threshold corresponds to a pair of coordinates for the false positive rate versus the true positive rate. In Figure 5, the *x*-axis represents recall, and the *y*-axis represents precision at different thresholds of the anomaly scores. Each threshold corresponds to a pair of coordinates for recall versus precision. Other than the LOF model, both the AUC-ROC and the AUC-PR are very high for the remaining seven models. Among the seven models with strong performance, the AUC-ROC is ranged from 0.975 to 0.9954, and the AUC-PR is ranged from 0.9698 to 0.9946. The high AUC-ROC indicates a strong likelihood that the score of a randomly selected abnormal traffic record will be higher than that of a randomly selected normal traffic record, since higher scores tend to signal abnormal traffic. The high AUC-PR indicates that the model can accurately identify all intrusions without erroneously categorizing excessive normal traffic as intrusion. Compared to the other seven models, the LOF model performs relatively poorly with AUC-ROC of 0.716 and AUC-PR of 0.6219. However, it still outperforms the baseline, since a random guessing baseline model has an AUC-ROC of 0.5, and an AUC-PR equal to the prevalence of intrusions in the data. The LOF model exhibits AUC-ROC greater than 0.5 and AUC-PR higher than the intrusion prevalence, which stands at 0.4654 in the NSL-KDD dataset.

Both AUC-ROC and AUC-PR rank K-means as the best model and LOF as the worst model. For the rest models, the rank order differs slightly based on AUC-ROC and AUC-PR. In fact, the rank order of models based on AUC-ROC and AUC-PR is expected to differ, as they measure different aspects of model performance, particularly in imbalanced datasets where abnormal records are rare. AUC-ROC measures a model’s ability to distinguish between normal and abnormal classes across all possible thresholds, while AUC-PR summarizes how well a model maintains high precision at different recall levels. Since the number of intrusions and normal traffic records is comparable and there is no severe class imbalance issue in the NSL-KDD dataset, the rank order based on AUC-ROC and AUC-PR only differs slightly.

**Figure 6:**
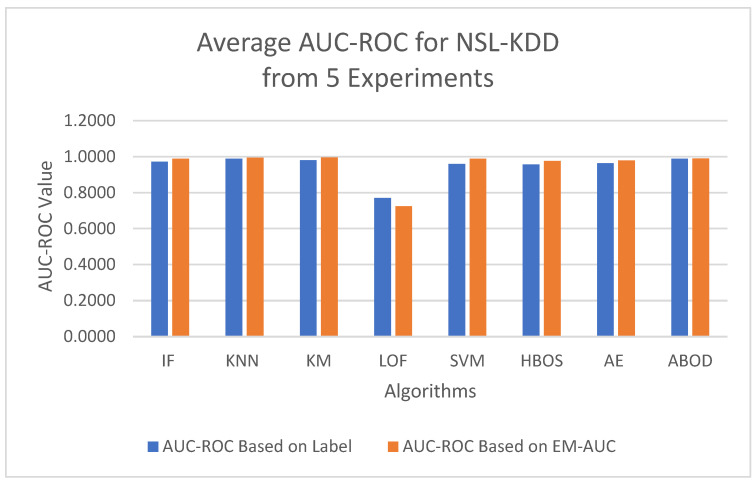
Average AUC-ROC Comparison: EM-AUC vs. Imperfect Labels (NSL-KDD).

**Figure 7:**
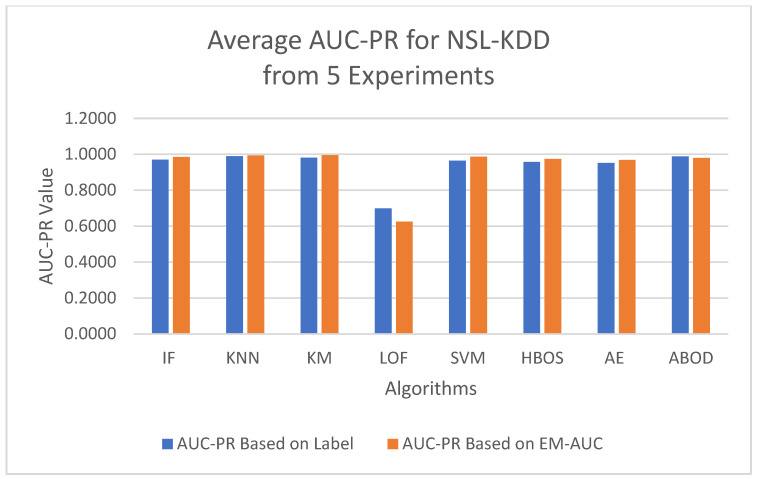
Average AUC-PR Comparison: EM-AUC vs. Imperfect Labels (NSL-KDD).

To minimize bias from a single random data split, the AUC-ROC metric was averaged across five different test sets for each model. The first column in Table 4 contains the average AUC-ROC estimated using the EM-AUC algorithm for each model. For comparison purposes, the second column in Table 4 contains the average AUC-ROC calculated based on the imperfect labels in the NSL-KDD dataset as described in Section 3.2.1, with the understanding that these labels are not the gold standard. Similarly, the AUC-PR metric was also averaged across five different test sets for each model, using either the EM-AUC algorithm or the imperfect labels, as summarized in Table 5. To assess whether the results produced by EM-AUC align with our expectations, we compared the AUC-ROC and AUC-PR derived from the proposed EM-AUC with those based on the labels in the NSL-KDD dataset. On average, the results based on the EM-AUC algorithm are very close to the ones based on the imperfect labels. As shown in Figure 6, the *x*-axis represents the unsupervised models, while the *y*-axis shows the average AUC-ROC value for each model across 5 experiments. The blue bars represent the average AUC-ROC based on labels, and the orange bars represent the average AUC-ROC based on EM-AUC. A similar bar chart was created for the average AUC-PR results from the 5 experiments as well in Figure 7. As shown in the bar charts, the EM-AUC algorithm tends to provide less conservative estimates of model performance compared to those based on imperfect labels, especially when the models outperform. In other words, model performance may be underestimated based on imperfect labels when the models perform better.

While the NSL-KDD dataset has been used as a benchmark to evaluate models for intrusion detection, it is recognized to have certain shortcomings. There is no exact definition of the attacks in the NSL-KDD dataset. The labels are considered as an imperfect standard. For example, probing may be normal or may be a precursor to some other activities. Probing is not necessarily an attack type unless the number of iterations exceeds a specific threshold. Similarly, a packet that causes a buffer overflow is not always representative of an attack [49]. When an imperfect standard or label is used as if it were a gold standard, the performance of the model is often either underestimated or overestimated. The EM based algorithm is known to effectively capture data patterns, especially in cases where the data follows a mixture model with subgroups. In situations where the gold standard is not perfect or there is uncertainty in the labels, simulation studies show the EM based algorithm produces a more robust estimation of performance metrics compared to directly relying on the imperfect standard [1].

#### 4.3.2. UNSW-NB15 Results

**Figure 8:**
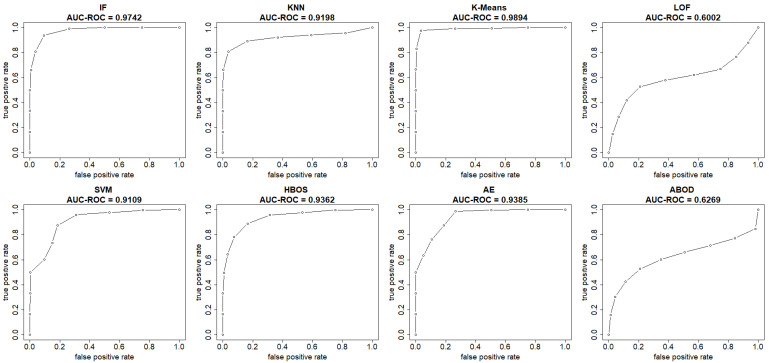
ROC curves (FPR vs. TPR) estimated by EM-AUC for UNSW-NB15 dataset from one experiment.

**Figure 9:**
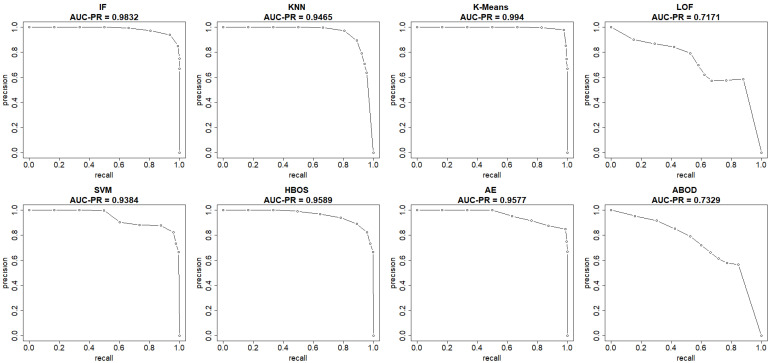
Precision-Recall curves (recall vs. precision) estimated by EM-AUC for UNSW-NB15 dataset from one experiment.

This section summarizes the results from the experiments on the UNSW-NB15 dataset. The plots in Figure 8 and Figure 9 show both the ROC and precision-recall curves, estimated using the EM-AUC algorithm, for each unsupervised learning model in one of the experiments. In Figure 8, the *x*-axis represents the false positive rates, and the *y*-axis represents the true positive rates at different thresholds of the anomaly scores. Each threshold corresponds to a pair of coordinates for the false positive rate versus the true positive rate. In Figure 9, the *x*-axis represents recall, and the *y*-axis represents precision at different thresholds of the anomaly scores. Each threshold corresponds to a pair of coordinates for recall versus precision. All six models, excluding LOF and ABOD, achieve high AUC-ROC and AUC-PR, with AUC-ROC ranging from 0.9109 to 0.9894 and AUC-PR from 0.9384 to 0.994. In contrast, LOF and ABOD perform relatively poorly but still better than the baseline. LOF has an AUC-ROC of 0.6002, exceeding the baseline of 0.5, and an AUC-PR of 0.7171, which is higher than the intrusion prevalence of 0.6806 in the UNSW-NB15 dataset. Similarly, ABOD has an AUC-ROC of 0.6269 and an AUC-PR of 0.7329, both surpassing baseline performance. K-means ranks as the best model, and LOF as the worst, which is consistent with the observations from the NSL-KDD dataset.

**Figure 10:**
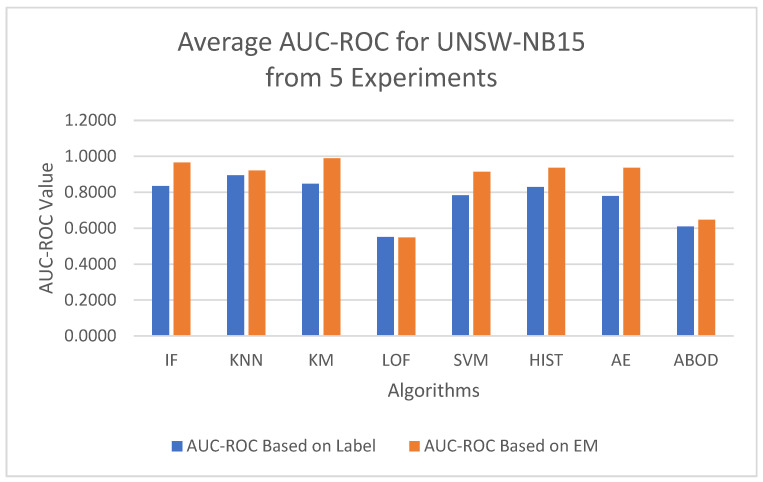
Average AUC-ROC Comparison: EM-AUC vs. Imperfect Labels (UNSW-NB15).

**Figure 11:**
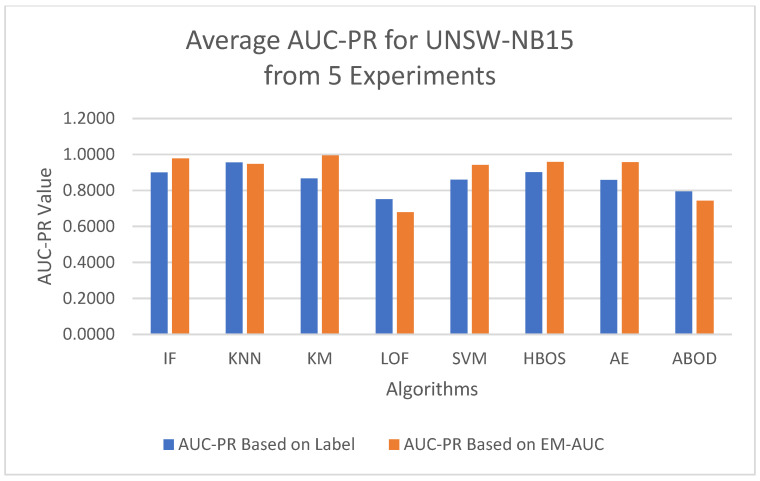
Average AUC-PR Comparison: EM-AUC vs. Imperfect Labels (UNSW-NB15).

To minimize bias from a single random data split, the AUC-ROC was computed as the average across five distinct test sets for each model, based on either the EM-AUC algorithm or the imperfect labels, as detailed in Table 6. Similarly, the AUC-PR was averaged over these five test sets for each model, using either the EM-AUC algorithm or the imperfect labels, as summarized in Table 7. To assess whether the results produced by EM-AUC align with our expectations, we compared the AUC-ROC and AUC-PR derived from the proposed EM-AUC with those based on the labels in the UNSW-NB15 dataset. On average, the AUC-ROC and AUC-PR measurements from the EM-AUC algorithm and those from the imperfect labels show a comparable rank order. Model LOF was ranked as the least performing model by all metrics, either based on imperfect labels or the EM-AUC algorithm. KNN was ranked as the best model by AUC-ROC and AUC-PR based on imperfect labels, while K-means was ranked as the best model by AUC-ROC and AUC-PR based on the EM-AUC algorithm. For most models, the EM-AUC algorithm tends to provide more optimistic estimates of model performance compared to the results based on imperfect labels as illustrated in Figure 10 and Figure 11.

Both the NSL-KDD dataset and the UNSW-NB15 dataset were generated using simulated labeling in an artificial network environment. While simulated labeling methods efficiently produce a significant amount of labeled network traffic, the accuracy of these labels is not guaranteed. The performance of a model can be either underestimated or overestimated if an inaccurate label is used. As observed in this study, model performance based on the labels in the NSL-KDD dataset is better than that based on the labels in the UNSW-NB15 dataset, indicating that the quality of the NSL-KDD dataset is higher. The NSL-KDD dataset is considered an improved version of the original KDD99 dataset, addressing several inherent issues, such as mislabeling and the poor definition of certain attack types [50]. In contrast, there is no improved version of the UNSW-NB15 dataset that incorporates additional quality controls. As a result, discrepancies between model performance based on the labels and those derived from the EM-AUC algorithm are more pronounced in the UNSW-NB15 dataset compared to the NSL-KDD.

## 5. Conclusions

We introduced a novel system that leverages the EM-AUC algorithm to derive AUC-ROC and AUC-PR for evaluating the performance of anomaly scores produced by unsupervised machine learning models. Both unsupervised learning models and the EM-AUC algorithm do not require comprehensive labels. To the best of our knowledge, this study is the first to utilize the EM-AUC algorithm for evaluating unsupervised learning models for network intrusion detection. The original approach proposed by Zhou et al. [1] provides only the AUC-ROC metric. In our study, we expand its capability to also estimate additional performance metrics, such as AUC-PR, alongside AUC-ROC.

The key contribution of our research is the development of an algorithm that enables performance evaluation without relying on labels. The EM-AUC algorithm provides robust performance estimates in situations where labels are unavailable or flawed. The label-free, algorithmically derived AUC-ROC and AUC-PR offer a more comprehensive assessment by summarizing false positive rates, true positive rates, precision, and recall across all thresholds along the ROC and precision-recall curves, rather than just a single set of values at one threshold. Additionally, the AUC-PR derived from the EM-AUC method better assesses model performance when class imbalance issues are present. Since training, testing, and evaluating the unsupervised models do not require a comprehensively labeled dataset in the proposed system, it offers a cost-effective and scalable solution for network intrusion detection without the need to label or update massive volumes of network traffic data. When true labels are unavailable, the unsupervised model with the highest AUC-ROC or AUC-PR can be selected using the EM-AUC algorithm. The final threshold for the anomaly scores of the selected model should be set by balancing the risks of false positives and false negatives, tailored to the specific operational needs of network intrusion detection.

## 6. Discussion and Future Studies

In this study, we apply our proposed approach, EM-AUC, to evaluate unsupervised models. EM-AUC is model-agnostic and can assess the performance of any model—supervised or unsupervised—when labels are unavailable or unreliable, as long as the model provides a probability or score indicating the likelihood of anomaly for each data point. This anomaly score or probability is then fed into the EM-AUC algorithm, which generates performance metrics without requiring labels.

There are several potential directions for further research. One promising direction is to develop an ensemble method that synthesizes labels for anomaly detection. We plan to incorporate an additional optimization algorithm to assign weights to different unsupervised algorithms based on their estimated performance, as evaluated by the EM-AUC approach. The optimal cutoff point of the composite anomaly scores from the ensemble can then be determined to produce synthetic labels. We anticipate that this ensemble approach for producing synthetic labels will lead to more accurate anomaly detection.

We also plan to extend the use of EM-AUC to evaluate alternative supervised models, particularly when labels are flawed. For real-world detection, the EM-AUC algorithm can compute AUC-ROC and AUC-PR, enabling continuous monitoring of a model’s performance on incoming, unlabeled network traffic data. Additionally, we aim to estimate standard errors and confidence intervals for AUC-ROC and AUC-PR using the EM-AUC algorithm. This will provide a more comprehensive view of model performance, helping users understand the potential variability in the results. Finally, we plan to expand our proposed system to other domains, such as malware and fraud detection.

## Figures and Tables

**Table 1:** Confusion matrix.

Total = P+N		Predicted Outcome	
		Positive (PP)	Negative (PN)
**Actual Outcome** **(i.e., Perfect Label)**	Positive (P)	True Positive (TP)	False Negative (FN)
	Negative (N)	False Positive (FP)	True Negative (TN)

**Table 2:** KDDTrain+ dataset summary.

Label	No. of Records
Normal	67,343
Attack	58,630
Total	125,973
Intrusion Prevalence	0.4654

**Table 3:** UNSW_NB15_training-set summary.

Label	No. of Records
Normal	56,000
Attack	119,341
Total	175,341
Intrusion Prevalence	0.6806

**Table 4:** Average AUC-ROC metrics based on five experiments for NSL-KDD dataset.

Model	AUC-ROC Based on EM-AUC	AUC-ROC Based on Imperfect Label
IF	0.9885	0.9724
KNN	0.9947	0.9891
KM	0.9954	0.9799
LOF	0.7243	0.7699
SVM	0.9888	0.9599
HBOS	0.9763	0.9567
AE	0.9795	0.9637
ABOD	0.9898	0.9892

**Table 5:** Average AUC-PR metrics based on five experiments for NSL-KDD dataset.

Model	AUC-PR Based on EM-AUC	AUC-PR Based on Imperfect Label
IF	0.9856	0.9696
KNN	0.9939	0.9889
KM	0.9946	0.9807
LOF	0.6252	0.6982
SVM	0.9871	0.9647
HBOS	0.9745	0.9570
AE	0.9691	0.9525
ABOD	0.9793	0.9878

**Table 6:** Average AUC-ROC metrics based on five experiments for UNSW-NB15 dataset.

Model	AUC-ROC Based on EM-AUC	AUC-ROC Based on Imperfect Label
IF	0.9658	0.8351
KNN	0.9209	0.8952
KM	0.9896	0.8466
LOF	0.5483	0.5517
SVM	0.9146	0.7829
HBOS	0.9361	0.8295
AE	0.9366	0.7789
ABOD	0.6470	0.6099

**Table 7:** Average AUC-PR metrics based on five experiments for UNSW-NB15 dataset.

Model	AUC-PR Based on EM-AUC	AUC-PR Based on Imperfect Label
IF	0.9772	0.9001
KNN	0.9471	0.9557
KM	0.9941	0.8669
LOF	0.6790	0.7511
SVM	0.9410	0.8594
HBOS	0.9585	0.9010
AE	0.9564	0.8585
ABOD	0.7421	0.7941

## Data Availability

NSL-KDD dataset: https://www.kaggle.com/datasets/hassan06/nslkdd (accessed on 18 March 2024). UNSW-NB15 dataset: https://research.unsw.edu.au/projects/unsw-nb15-dataset (accessed on 18 March 2024).
